# Diagnostic accuracy of telemedicine for detection of surgical site infection: a systematic review and meta-analysis

**DOI:** 10.1038/s41746-022-00655-0

**Published:** 2022-08-03

**Authors:** Ross Lathan, Misha Sidapra, Marina Yiasemidou, Judith Long, Joshua Totty, George Smith, Ian Chetter

**Affiliations:** 1 Centre for Clinical Sciences, Hull York Medical School, Hull, UK; 2 Bradford Teaching Hospitals, Bradford, UK; 3 Department of Plastic and Reconstructive Surgery, Hull University Teaching Hospitals NHS Trust, Cottingham, UK

**Keywords:** Diagnostic markers, Outcomes research

## Abstract

The Sars-CoV-2 pandemic catalysed integration of telemedicine worldwide. This systematic review assesses it’s accuracy for diagnosis of Surgical Site Infection (SSI). Databases were searched for telemedicine and wound infection studies. All types of studies were included, only paired designs were taken to meta-analysis. QUADAS-2 assessed methodological quality. 1400 titles and abstracts were screened, 61 full text reports were assessed for eligibility and 17 studies were included in meta-analysis, mean age was 47.1 ± 13.3 years. Summary sensitivity and specificity was 87.8% (95% CI, 68.4–96.1) and 96.8% (95% CI 93.5–98.4) respectively. The overall SSI rate was 5.6%. Photograph methods had lower sensitivity and specificity at 63.9% (95% CI 30.4–87.8) and 92.6% (95% CI, 89.9–94.5). Telemedicine is highly specific for SSI diagnosis is highly specific, giving rise to great potential for utilisation excluding SSI. Further work is needed to investigate feasibility telemedicine in the elderly population group.

## Introduction

Surgical site infections (SSI) complicate up to 40% of surgical procedures depending on operative type and procedure^1^. By definition, an SSI occurs within 30 days of surgery (or within 90 days if an implant is left in place)^2^. Given the current health landscape, the mean postoperative inpatient stay is four days, therefore the majority of SSI become apparent after discharge^3,4^. Early recognition of SSI is essential to minimise associated morbidity and mortality, and patients frequently seek care from primary or community care providers, who may not be familiar with managing surgical complications. Strategies are required to enable secondary care providers to conduct robust surveillance and follow up of surgical wounds^5,6^.

Telemedicine is an innovative solution for monitoring patients and their wounds postoperatively. Remote consultations ameliorate the need to leave home and associated carer requirement, reduce travel times and costs, and reduce waiting room times and risk of nosocomial infection^7–9^. Patients frequently find the experience reassuring and many would prefer future consultations by this method^10–12^. A reduction in patient travel seems to have wider implications still; a recent review concluded that the use of telemedicine consistently reduces carbon footprint compared with face-to-face reviews, even when factoring the impact of equipment and resource use^13^. With national targets such as net zero emissions by 2045, implementation of remote measures may become a mainstay of practice in years to come^14^. The SARS-CoV-2 pandemic catalysed integration of digital health models worldwide and telemedicine was applied at the forefront of many patient facing services. Surgical follow-up has followed with rapid adoption of remote post-operative follow-up, but cautious examination is warranted before being welcomed as standard practice^15^. Telephone consultations, whilst providing invaluable information at a fraction of clinic resource use, do not provide direct visualisation of a patient’s post-operative wound. However, even with the addition of a visual aspect in photo- or video-based approaches, there are barriers to this service. For example, erythema, a hallmark characteristic of accepted SSI definitions, has poor levels of interobserver agreement on photograph assessment^14,16,17^. Before telemedicine can be unanimously recognised as established practice substantial evidence of diagnostic accuracy is required.

The aim of this study was to (1) establish the overall accuracy of telemedicine for diagnosis of SSI; (2) identify factors associated with heterogeneity of findings between studies; and (3) assess the effect of individual telemedicine methods and impact of varying reference standards on diagnostic accuracy.

## Results

### Study selection

**Fig. 1:**
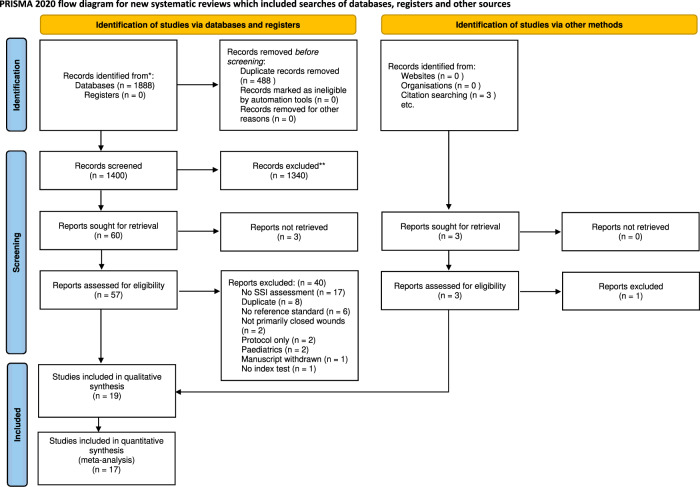
PRISMA flow diagram for study selection process. Identification of studies via databases and registers is found in the left column, and via other methods on the right.

The study selection process flow diagram is shown in Fig. 1. A total of 1400 records were screened after 488 duplicates removed. After title and abstract screening, 61 full text reports were assessed for eligibility. The final review included 19 studies, and 17 had paired designs taken into a meta-analysis^16,18–35^. 11,437 observations were made in 19,090 patients as ten studies only included telemedicine investigation in a subset of patients^18–22,25,26,28,30,32^. Three reports were unable to be retrieved. For each, contact was attempted through the publishing journal and first author on two separate occasions, after which studies were excluded from review.

### Characteristics of included studies

Studies were conducted in nine countries across five continents globally. Five were in low or lower-middle income economies, as per the World Bank classification^18,19,22,27,28^. The remaining reports were from high income economies. Weighted mean age of participants across the included reports was 47.1 ± 13.3 years. Female patients made up 57.4% participants. Pooled SSI rate was 5.6% (95% CI, 5.49–5.74). Individual study characteristics can be found in Table 1.

**Table 1:** Individual study characteristics.

Author	Year	Country	Mean Age (±SD)	Total events	Total Patients	Female Gender (%)	Telemedicine method	Reference standard	Type of surgery	SSI review provider	Diabetes (%)	Smoking (%)	BMI (± SD)
Abu-sheasha	2020	Egypt	—	309	351	—	Telephone	CDC	Multispecialty	Healthcare worker	—	—	
Aiken	2013	Kenya	30.0 (9.0)	89	1172	1060	Telephone	CDC	Multispecialty	Healthcare worker	—	—	—
Bluebelle	2019	UK	53.2 (17.5)	208	732	428	Questionnaire	CDC	Multispecialty	Research Team	60 (7.7)	350 (45.1)	28.0 (6.1)
Bruce	2021	UK	44.1 (14.1)	1036	1550	302	Photograph	CDC	Trauma	Surgeon	63 (8.1)	218 (28.6)	26.4 (5.9)
Cherian	2020	Rwanda	26.5	219	596	456	Telephone	Empirical	O&G	Community healthcare worker	—	—	—
Gunter	2018	USA	63.0	40	40	10	Photograph	Site protocol	Vascular	Clinical or research team	—	—	—
Halwani	2016	USA	28.5 (6.8)	177	193	193	Telephone	CDC	O&G	—	—	—	33.3 (9.2)
Hedrick	2015	USA	59.5 (6.7)	171	171	89	Photograph	CDC	General	Surgeon	21 (12.3)	28 (16.4)	29.0 (7.5)
McLean	2021	UK	44.3 (17.3)	335	489	266	Photograph	CDC	General	Surgeon	23 (4.7)	—	24.2
Mitchell	1999	Australia	63.3	649	1360	636	Questionnaire	CDC	Multispecialty	Research nurse	—	—	—
Mousa	2019	USA	64.0 (7.2)	30	30	14	Photograph		Vascular	—	12 (40.0)	15 (50.0)	28.4 (5.6)
Nguhuni	2017	Tanzania	26.3 (6.5)	484	324	324	Telephone	CDC	Obstetrics	Surgeon / Research nurse	—	—	26.1 (5.0)
Pathak	2015	India	—	156			Telephone	CDC	—	‘Study assistant’	—	—	—
Pham	2016	USA	54.1(19.0)	2853	2853	1844	Telephone	CDC	Multispecialty	Surgeon	367 (12.9)	359 (12.6)	29.5 (7.4)
Reilly	2005	UK	67.0 (10.4)	105	422	201	Telephone	CDC	Orthopaedics	GP / district nurse	—	—	—
Richter	2017	Israel	50.5 (17.7)	263	266	125	Telephone	CDC	General	Surgeon (face-to-face) / ‘Surveyor’ (telephone)	—	—	—
Sands	1996	USA	42.0	1799	5572	3343	Questionnaire	CDC	Multispecialty	Record reviewer	—	—	—
Taylor	2003	UK	—	2665	—		Telephone	CDC	General	Healthcare worker	—	—	—
Totty	2018	UK	61.1	56	37	14	Photograph	ASEPSIS/CDC	Vascular	Surgeon	10 (27.0)	28 (75.7)	—

*SD* Standard Deviation; *BMI* Body mass index.

### Methodological quality of included studies

**Fig. 2:**
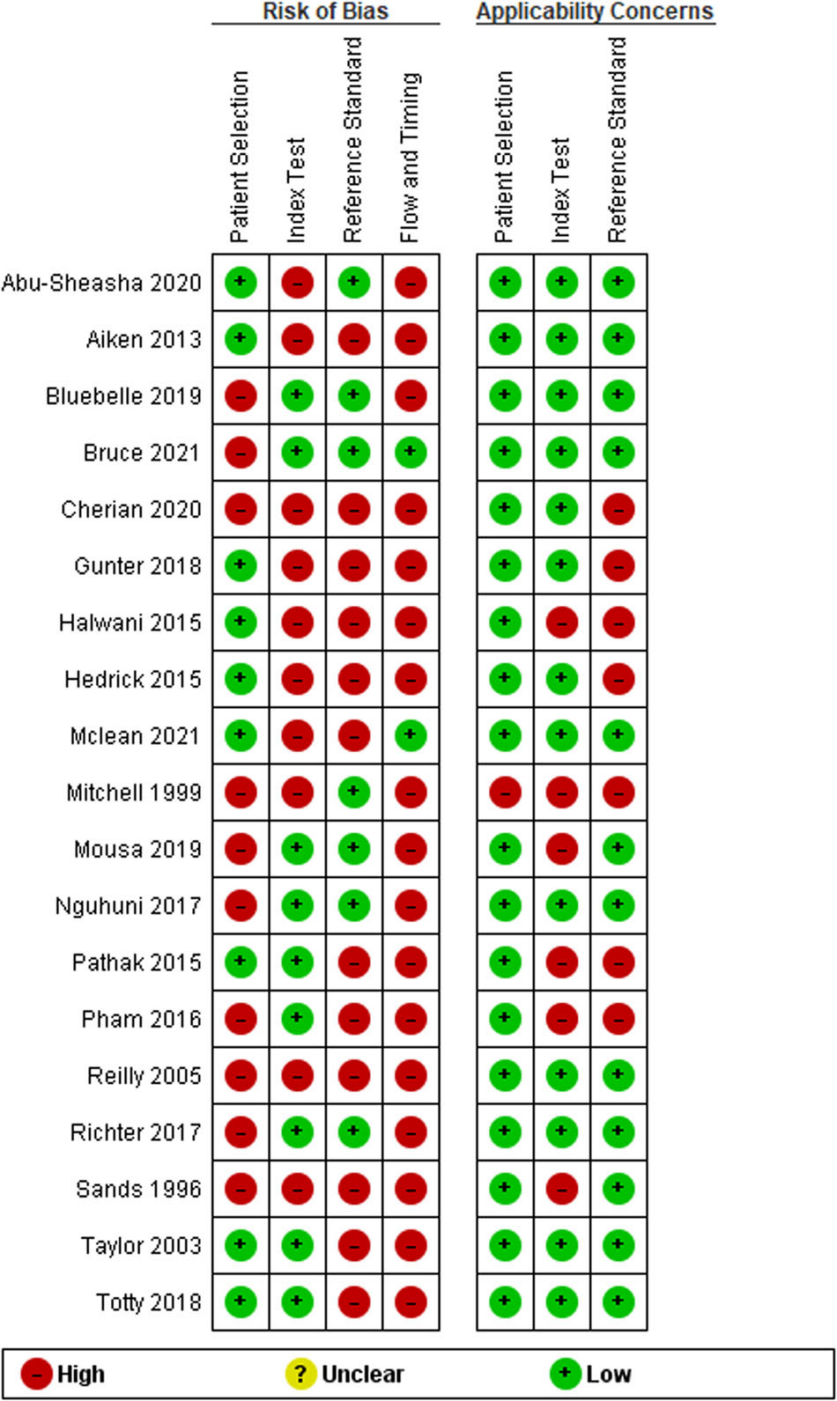
Risk of bias and applicability concerns summary: review authors’ judgements about each domain for each study. High, unclear and low risk of bias and applicability concerns are represented as shown in the legend.

**Fig. 3:**
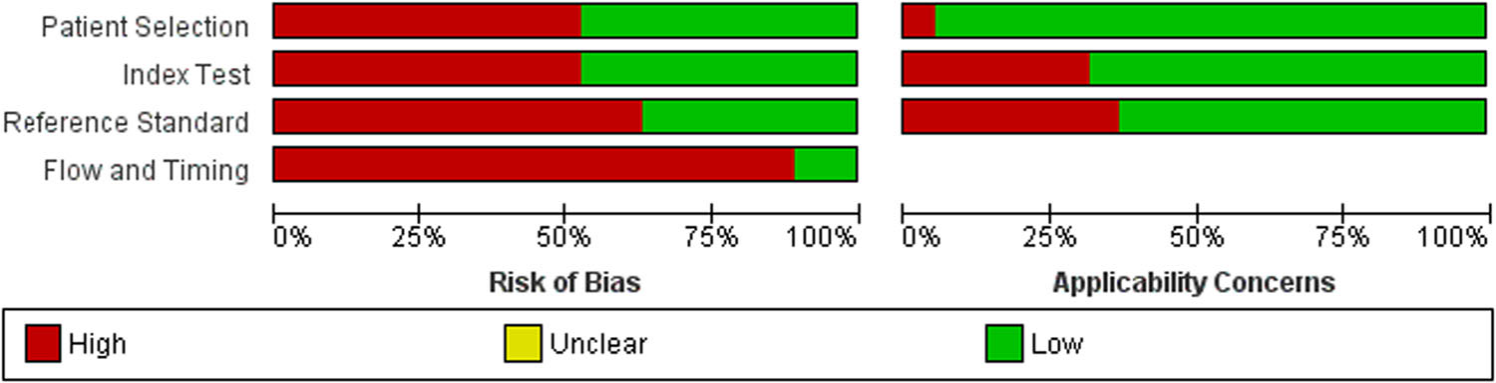
Risk of bias and applicability concerns graph: review authors’ judgements about each domain presented as percentages across included studies. High, unclear and low risk of bias or applicability concerns are represented as shown in the legend.

A summary of QUADAS-2 assessments is presented in Fig. 2. Risk of bias was present in all studies, and two studies were scored as high risk of bias in all domains^22,30^. This was largely owing to inconsecutive sampling, clarity over interpretation of index tests without knowledge of the reference standard (and vice versa), the interval time between interpretation of index test and reference standard, and only subgroups of patients being included in study analysis. Nine reports had high applicability concerns, principally from the index test, patients or reference standard differing from the review question^22–24,26,28,29,32,34^. Risk proportions are displayed in Fig. 3.

### Synthesis of results

**Fig. 4:**
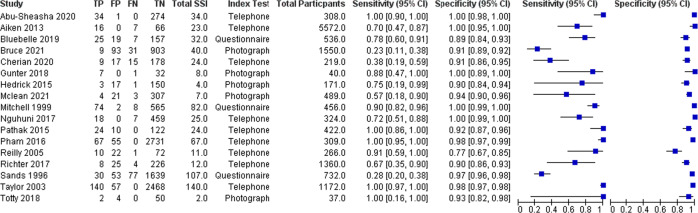
Coupled forest plot presenting sensitivity and specificity of SSI diagnosis by telemedical methods. Final two columns display the sensitivity and specificity respectively with 95% confidence intervals.

Individual study estimates of test accuracy are presented in Fig. 4 as a coupled forest plot of sensitivity and specificity. Index tests were categorised into photograph, telephone and questionnaire based methods, with five^16,21,23–25^, nine^18,19,22,27–31,33^ and three studies^20,26,32^ available for each respectively. 15 manuscripts^16,18–21,24–33^ utilised a CDC based reference standard, with the remaining two^22,23^ having empirical or site-specific protocol for these. Two studies^22,23^ conducted follow up within 14 days, and a further four studies^24,26,30,32^ were unclear as to the timeframe for reference standard review. There were no studies available that compared multiple index tests or reference standards.

**Fig. 5:**
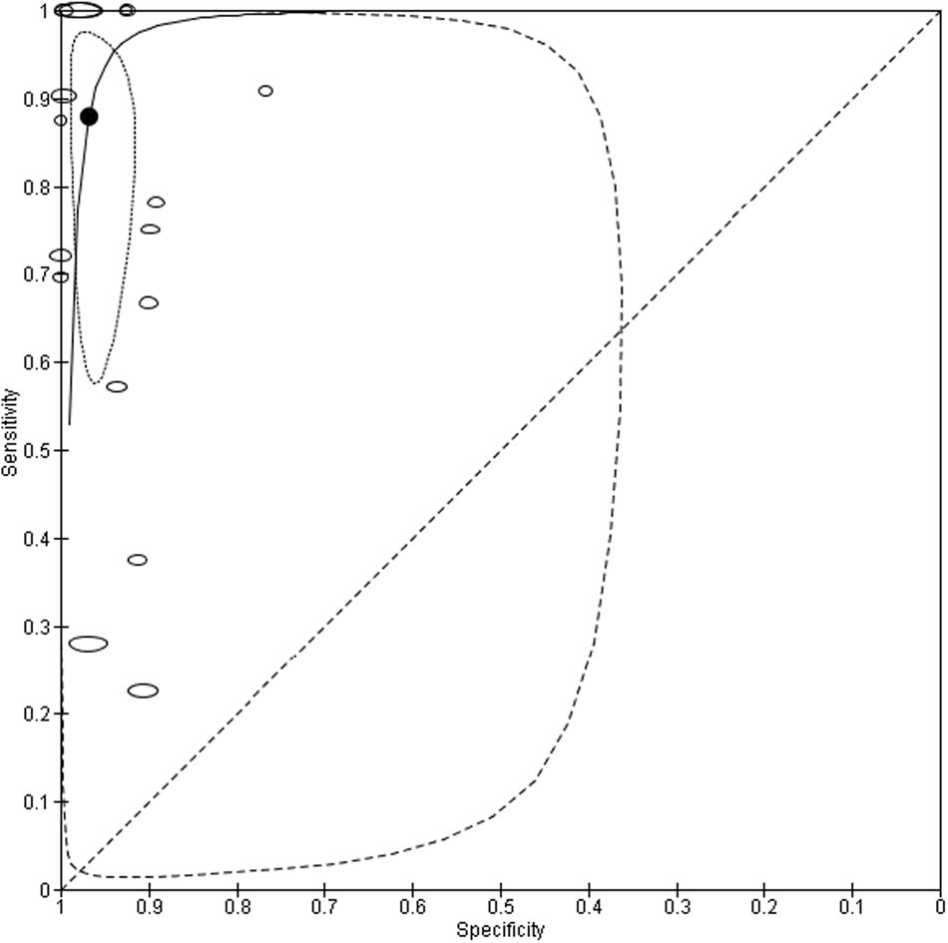
Random effects bivariate summary receiver operator characteristic curve of telemedicine for the diagnosis of surgical site infection. Summary curve and point estimates display high levels of accuracy. Elliptical data points represent weight sensitivity-specificity trade-off for each study. The summary point is expressed in the summary curve with dotted line 95% confidence region and dashed line 95% prediction region.

The mean sensitivity of all telemedical methods for SSI diagnosis is 87.9% (95% CI, 68.4–96.1) and mean specificity is 96.8% (95% CI, 93.5–98.4). Mean values broken down by index test is shown in Table 2. Youden’s index is acceptable at 0.847. The mean positive and negative predictive values for diagnosis of SSI are 54.8% (95% CI, 52.1–57.4) and 98.5% (95% CI, 98.2–98.6) respectively. Random effects SROC curve for all methods of telemedicine in diagnosis of SSI shows a symmetric design approaching the top left corner and is plotted in Fig. 5. Heterogeneity seen in the 95% prediction region is explored further in subgroup analysis. Overall diagnostic odds ratio indicates high effectiveness for SSI diagnosis at 217.6 (95% CI, 47.0–1006.8).

**Table 2:** Summary test accuracy of surgical site infection diagnosis by index test method.

Index Test	Reference Standard	Number of Studies (Participants)	Number with SSI (%)	Summary sensitivity % (95% CI)	Summary Specificity % (95% CI)	Diagnostic Odds Ratio (95% CI)
All	All	17 (11437)	642 (5.6)	87.9 (68.4–96.1)	96.8 (93.5–98.4)	217.6 (47.0–1006.8)
Photograph	All	5 (1638)	61 (3.7)	63.9 (30.4–87.8)	92.6 (89.9–94.5)	22.0 (4.7–102.5)
Telephone	All	9 (7143)	360 (5.0)	97.0 (70.8–99.8)	97.7 (92.0–99.4)	1351.5 (73.1–24994.7)
Questionnaire	CDC	3 (2656)	221 (8.3)	69.8 (32.6–91.7)	97.6 (88.7–99.5)	93.8 (6.4–1366.8)

*SSI* surgical site infection; *CI* confidence interval.

### Subgroup analysis

**Fig. 6:**
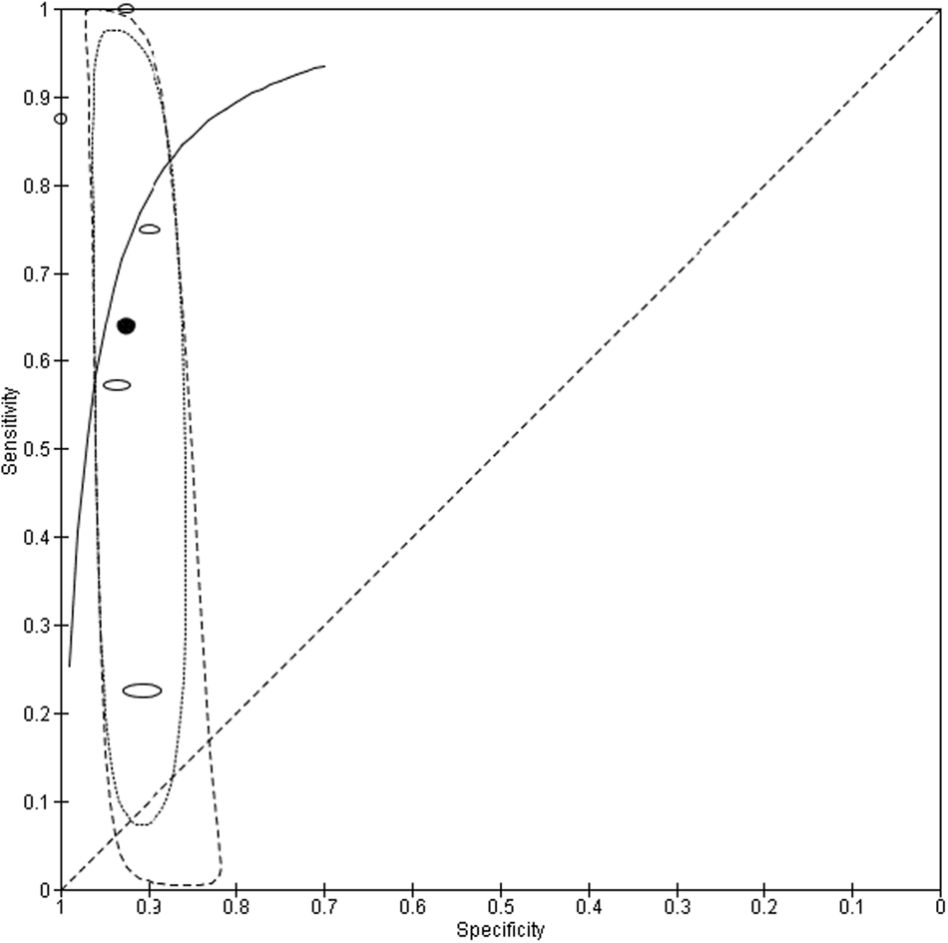
Random effects bivariate summary receiver operator characteristic curve for photograph based recognition of surgical site infection. Elliptical data points represent weight sensitivity-specificity trade-off for each study. The summary point is expressed in the summary curve with dotted line 95% confidence region and dashed line 95% prediction region.

Five studies utilised photograph based telemedicine^16,21,23–25^. Two studies^16,21^ retrieved images with a digital camera, another two^23,25^ used smartphone and a final study^24^ did not specify the platform used. No studies used machine learning or ‘artificial intelligence’ methods to assist in diagnosis of SSI. A total of 1638 observations were available in 2287 patients, again due to subsets being included in diagnostic test accuracy analysis. The weighted average age was 46.8 ± 11.7 years and 35.8% of patients were female. All studies were conducted in high income countries (HIC). SSI rate across the available studies was 3.72% (95% CI, 3.16–4.29). The mean sensitivity for photograph based methods is 63.9% (95% CI, 30.4–87.8) and mean specificity 92.6% (95% CI, 89.9–94.5). The mean positive and negative predictive values for SSI diagnosis are 15.6% (95% CI, 11.6–20.7) and 97.6% (95% CI, 97.0–98.0). The random effects SROC curve for photograph based methods shows a symmetric distribution and is displayed in Fig. 6. Overall diagnostic odds ratio indicates good test effectiveness at 22.0 (95% CI, 4.7–102.5). Heterogeneity is largely reduced, although the region of confidence is conversely enlarged.

Comparative index test SROC curve analysis reveals three distinct distributions of symmetric plots with telephone methods showing superior test accuracy, approaching the upper left corner (Supplementary Fig. 1). CDC criteria were used as the reference standard in all but two studies^22,23^. Analysis of tests standardised by CDC reference standard marginally increases overall sensitivity to 90.3% (95% CI, 0.695–0.974) but has no significant impact on specificity (96.8%, 95% CI, 0.932–0.985). SROC curve for telemedicine using CDC criteria reflects this marginal increase in sensitivity but 95% prediction region is also increased in size (Supplementary Fig. 2). Summary test accuracy by CDC reference standard is represented in Supplementary table 2. All methods of telemedical follow-up are informative with diagnostic odds ratios >10. Five studies diagnosed SSI through review by surgeons. Observations from 4451 participants were available resulting in lower overall sensitivity at 84.5% (95% CI, 22.3–99.0) and specificity at 94.5% (95% CI, 90.9–96.8). Heterogeneity in diagnosis was reduced with this limitation but at the cost of a greater 95% confidence region.

## Discussion

Telemedicine achieves good sensitivity (88%) and high specificity (97%) irrespective of geographical location and socioeconomic status. As such, remote methods could be considered globally as a screening tool for SSI post discharge, on the basis that correctly identifying patients without an infection can prevent them from needing to travel long distances to see a clinician, and conversely those diagnosed as having had infection can be signposted to appropriate post-operative care at an earlier stage. Telephone-based appears to be the most accurate telemedicine method in SSI diagnosis, and has been the most extensively studied index test in the last two decades. Arguably this is the most readily deployable and economically viable option. Telephone discussions enable real-time data collection from the patient with follow-up questions adaptable to the scenario, and conversely, can be used to deliver validated questionnaires by untrained individuals, should the need arise (as part of widespread screening, for example). Clinicians can obtain further information from patients in response to signalling, such as exploring systemic symptoms of infection, that may not be derived through images alone.

In contrast, photograph-based methods are contemporary, with all studies conducted within the last seven years. This index test offers additional visual stimuli which other methods do not, debatably of paramount importance in the diagnosis of SSI. Unlike telephone methods, photograph reviews are asynchronous, with data collected at a time prior to their review, preventing flexible further questioning. There may be a lack of standardisation across photograph based studies as image quality and provision of wound photography guidance (or training) are important factors in determining test accuracy, however these elements were not extractable from the available literature and may have contributed to the lower accuracy compared with telephone based methods^36^. In addition, diagnosis of SSI based on appearance alone is subjective, whether in person or using digital images, and this subjectivity may reduce the overall accuracy of digital assessment. Standardisation of wound photograph technique, and patient acceptability in digitally naïve populations (due to both age and socioeconomic status) are important research factors to be established in this area. Further, no single study investigated more than one diagnostic method or the impact on test accuracy with a combination of techniques i.e. photograph review with simultaneous questionnaire submission or with telephone review for concurrent data extraction, or the impact of video based assessments, where dialogue and a contemporaneous wound assessment can take place. Future studies should assess the diagnostic accuracy of combined or novel telemedicine methods in order to determine the optimal approach.

Postoperative wound surveillance is notoriously challenging, resulting in underreporting of SSI rates^37,38^. Overstretched primary care services are often burdened with facilitating management of such complications after discharge. Digital telemedicine requires limited resources and has potential utility in alleviating primary care exigency by offering a direct connection with secondary or tertiary care providers. Healthy wounds can be easily identified without the inconvenience of travelling great distances to clinic. Equally, obvious and indeterminate infection can be swiftly identified and either appropriately managed remotely or returned to secondary care for definitive treatment.

The National Health Service (NHS) in the UK is committed to delivering a net zero service by 2045^14^. In the NHS in England, up to 10% of total carbon dioxide emissions are attributable to personal travel, and specifically, emissions from patient travel have almost doubled since 1990 (0.63 to 1.23 Mt CO_2_e)^39^. Each hospital outpatient appointment is estimated to produce 76 kg CO_2_e, and a visit to general practice produces an estimated 66 kg CO_2_e^39^. Digital, remote follow-up offers some mitigation in the personal travel targets for a greener NHS. Further, artificial intelligence, or machine learning has been identified as a route to buttress the emission reduction effort^40^. If established into practice, machine learning could reasonably be applied to digital wound surveillance models to alleviate clinician time, minimise carbon footprint and unburden clinical resource use.

The telemedicine population studied is relatively young (weighted mean average age 47.1 years) which may reflect usability and is unlikely to represent the entire surgical population. Vascular patients for instance, are frequently much older (average age 64.1 years) and comorbid, as such may not be able to comply with smartphone-based telemedicine methods^41^. Investigation of telemedicine in elderly patients has identified a lack of access and experience with technology, and hearing, visual and communication challenges as barriers to utilisation^42,43^. Widespread adoption of such strategies without efforts to improve inclusion may be disproportionately disadvantageous to the elderly or infirm population groups.

This study has some limitations. High levels of heterogeneity were apparent in the initial meta-analysis (Fig. 5), as expected from diagnostic test accuracy studies. Photograph based subgroup analysis provided moderation (Fig. 6), but substantially fewer observations (1638 compared with 11437 for all telemedicine) should warrant cautious interpretation, and were all from high income economies. All included studies contained high risk of bias and so no exclusions were made on this basis alone. Most studies did not report test accuracy as their primary outcome and as such only subgroups of participants were included in analysis. No studies used a case-control design and one had a retrospective nature^32^. Three reports either had all or subsets of patients recruited in non-consecutive samples^20,26,32^. An unclear or inadequate (more than one week) time interval between index test reference standard was apparent in five studies^23,24,26,30,32^. Two studies investigated index tests or reference standards within two weeks of surgery^22,23^. The same reference standard was not used throughout, giving rise to potential for verification bias. Accuracy of diagnosis and heterogeneity did not however alter significantly in subgroup analysis using CDC criteria. Whilst recognised as the gold standard, CDC criteria is not without challenges. The classification is subjective and has poor interrater agreement, resulting in variable comparisons of wounds^44^. When compared with the ASEPSIS criteria, which are objective, ASEPSIS over classify SSI but under report SSI if pus is present^17,44,45^. The Southampton score is another alternative^45^. CDC is the most widely used, and as often regarded as the reference standard despite the inherent flaws. The need for a robust gold standard has been identified, but does not seem to have been accomplished yet.

The evidence suggests that using telemedicine, in the form of telephone consultation, with or without photographic adjuncts, to diagnose SSI is highly specific and as such could be utilised as an effective screening tool in patients post discharge. Implementation of this method has great potential in the reduction of resource use, associated healthcare cost, and patient and clinician time expenditure. It has widespread applications spanning geographical and socioeconomic barriers and would improve the carbon footprint of health services globally. However, the average age of participants in all studies is relatively young and as such may under-represent the surgical population. Widespread adoption of telemedicine without strategies to improve inclusion may therefore disproportionately discriminate against the elderly or infirm. Included studies were also at risk of bias which may impact upon the validity of results. Further work is required to maximise engagement with telemedicine in digitally naïve or incapable populations, and to determine the specific utility of telemedicine within clinical practice in order to maximise its benefits.

## Methods

This study was conducted in accordance with the Cochrane handbook for systematic reviews of diagnostic test accuracy, and has been reported in line with the Preferred Reporting Items for Systematic Reviews and Meta-Analyses (PRISMA-DTA) statement, a copy of which is attached to this article in the supplementary information (supplementary table 1)^46–48^. The protocol for this review was prospectively registered with PROSPERO (ID CRD42021290610) and has been submitted for peer reviewed publication, with a pre-print available online^49^.

### Search strategy and selection criteria

Studies meeting the following criteria were considered for inclusion:

i. Participants: All post-operative patients over 18, of any operation type. No restrictions were placed on the study setting or length of follow-up.
ii. Index tests: Telemedicine by any method (telephone, video call, photograph or questionnaire), including the use of questionnaires as these can be delivered remotely.
iii. Reference Standards: Face to face review, as per the United States (US) Centres for Disease Control and Prevention (CDC) criteria for SSI is deemed the gold standard, but no restrictions were placed if other methods were use. This was to ensure all available evidence would be synthesised.
iv. Target condition: SSI as defined by the CDC criteria; infection within 30 days of surgery or within 90 days if an implant is left in place^50^.
v. Study design: Abstracts, reviews and conference proceedings were excluded. All other research designs were included in the systematic review, but only comparative, paired methodologies were taken forward to meta-analysis as all patients would experience index tests and reference standards.

Studies were excluded if they did not meet the inclusion criteria or were not presented in English (for lack of resources to translate other languages). The following databases were searched from inception to January 2022: Medline, Embase, CENTRAL and CINAHL. A combination of synonyms related to the keywords; “telemedicine” AND “surgical wound infection” formulated the terms used. The strategy used for Medline, Embase and CINAHL can be found in supplementary information (supplementary methods 1).

The search strategy was developed with and conducted by an information specialist who uploaded results onto the Rayyan, a bespoke tool for conducting systematic reviews^51^. These were deduplicated before screening of titles and abstracts by two independent reviewers against the inclusion criteria. Relevant manuscripts were retrieved for full text review and assessed for eligibility by two independent reviewers. Reference lists of these articles were searched manually for any additional studies not identified in preliminary search. Any disagreement at each stage was resolved by a third reviewer for consensus.

There were no limitations placed on study design for qualitative synthesis to comprehensively synthesise the literature. Reports with paired designs were taken forward for quantitative analysis to enable random-effects bivariate meta-analysis, and summary receiver operator characteristic (SROC) curves to be plotted.

### Data extraction

A bespoke data spreadsheet (Microsoft Excel Version 16.59) was designed and utilised for data extraction by two independent authors. Data on study and diagnostic characteristics (author, year, country, study design, sample size, gender, age, telemedicine method, reference standard, type of surgery, follow-up schedule) among potential confounding factors (diabetes, BMI, and smoking status) were collected in addition to SSI rates, sensitivity, and specificity of diagnosis.

Surgical site infections were defined as per CDC criteria^2^. Only superficial SSI were included due to inherent barriers of diagnosing deep SSI remotely. No restrictions were placed on classification of telemedicine, reference standard type, or other characteristics.

### Assessment of methodological quality

Risk of bias and the applicability of studies were assessed again by two independent reviewers with the QUADAS-2 tool^52^. The tool was first piloted by the reviewers with agreement of 80% across all categories on two of the included studies considered sufficient before further assessment of remaining studies, as recommended by the Cochrane handbook for systematic reviews of diagnostic test accuracy^47^. QUADAS-2 contains four domains, each assessed for risk of bias; patient selection, index tests, reference standard and flow and timing. The first three domains are also investigated for applicability concerns. For each domain category, signalling questions are asked to assist judgments with answers ‘yes’, ‘no’ or ‘unclear’, such that ‘yes’ indicates low risk of bias. If any question is answered ‘no,’ this domain category is judged as high risk of bias or has applicability concerns. Answers of ‘unclear’ are used only if there was insufficient data reported. Risk of bias and applicability scores were taken into consideration for subgroup meta-analysis, ascertaining a strength of recommendation from data retrieved.

### Statistical analysis

Continuous descriptive characteristics were expressed as weighted mean averages with standard error. A bivariate model for meta-analysis was used to produce summary measures of sensitivity and specificity with confidence regions. All studies with paired designs had pooled forest plots and summary receiver operator characteristic curves synthesised in the initial exploratory analysis. Analysis was conducted with MetaDTA and plots constructed with Review Manager 5.4^53,54^. Additional sources of heterogeneity were investigated through covariates (study country, type of surgery, telemedicine method, reference standard used). Overall index test effectiveness is expressed through diagnostic odds ratios.

For cases of multi-threshold test positivity, the cut-off achieving the maximum possible sensitivity – specificity trade off were taken forward. Indeterminate index test results were classified as ‘no SSI’ as this more closely reflects what would happen in practice. Tests were grouped as a unified ‘telemedicine’ and through the sub-groups; ‘photograph,’ ‘telephone,’ and ‘questionnaire.’ No studies reported video-based methods.

### Subgroup analysis

All studies which compared photograph to face to face review will be referred to as photograph-based telemedicine methods. Photograph-based methods utilise visual input whereas questionnaire and telephone do not incorporate trained physicians viewing a patient’s wound. As such, pre-specified analysis is conducted for studies including these methods for their sensitivity and specificity. Further analyses are performed as per the reference standard used and whether a pre-specified threshold was stated.

### Reporting summary

Further information on research design is available in the Nature Research Reporting Summary linked to this article.

## Supplementary information

Supplementary Information

Reporting Summary

## Data Availability

The source data, and results of analysis, can be released upon reasonable request to the corresponding author.

## Code availability

No custom code or mathematical algorithms were used this study.

## References

[1] Matatov T, Reddy KN, Doucet LD, Zhao CX, Zhang WW (2013). Experience with a new negative pressure incision management system in prevention of groin wound infection in vascular surgery patients. J. Vasc. Surg..

[2] *Centres for Disease Control and Prevention (CDC). Surgical Site Infection (SSI) Event*, https://www.cdc.gov/nhsn/pdfs/pscmanual/9pscssicurrent.pdf (2017).

[3] Crothers, H. et al. Outcomes for surgical procedures funded by the English health service but carried out in public versus independent hospitals: a database study. *BMJ Quality Safety*. 10.1136/bmjqs-2021-013522 (2021). 10.1136/bmjqs-2021-013522 PMC9234423 34493605

[4] Woelber E., Schrick E. J., Gessner B. D. & Evans H. L. Proportion of surgical site infections occuring after hospital discharge: a systematic review. *Surgical Infections* **17**, 10.1089/sur.2015.241 (2016). 10.1089/sur.2015.241 27463235

[5] Lodise TP, McKinnon PS, Swiderski L, Rybak MJ (2003). Outcomes analysis of delayed antibiotic treatment for hospital-acquired Staphylococcus aureus bacteremia. Clin. Infect. Dis..

[6] Owens PL, Barrett ML, Raetzman S, Maggard-Gibbons M, Steiner CA (2014). Surgical site infections following ambulatory surgery procedures. Jama.

[7] Finkelstein SM (2012). Development of a remote monitoring satisfaction survey and its use in a clinical trial with lung transplant recipients. J. Telemed. Telecare.

[8] Gunter RL (2016). Current use of telemedicine for post-discharge surgical care: a systematic review. J. Am. Coll. Surg..

[9] Urquhart AC, Antoniotti NM, Berg RL (2011). Telemedicine-an efficient and cost-effective approach in parathyroid surgery. Laryngoscope.

[10] McGillicuddy JW (2013). Mobile health medication adherence and blood pressure control in renal transplant recipients: a proof-of-concept randomized controlled trial. JMIR Res Protoc..

[11] Wiseman JT (2015). Conceptualizing smartphone use in outpatient wound assessment: patients’ and caregivers’ willingness to use technology. J. Surg. Res.

[12] Sanger PC (2014). Patient perspectives on post-discharge surgical site infections: towards a patient-centered mobile health solution. PLoS ONE.

[13] Purohit A, Smith J, Hibble A (2021). Does telemedicine reduce the carbon footprint of healthcare? A systematic review. Future Health. J..

[14] NHS. *Delivering a ‘net zero’ National Health Service*, https://www.england.nhs.uk/greenernhs/wp-content/uploads/sites/51/2020/10/delivering-a-net-zero-national-health-service.pdf (2020).

[15] Irarrázaval MJ (2021). Telemedicine for postoperative follow-up, virtual surgical clinics during COVID-19 pandemic. Surg. Endosc..

[16] Totty JP, Harwood AE, Wallace T, Smith GE, Chetter IC (2018). Use of photograph-based telemedicine in postoperative wound assessment to diagnose or exclude surgical site infection. J. Wound Care.

[17] Wilson AP, Treasure T, Sturridge MF, Grüneberg RN (1986). A scoring method (ASEPSIS) for postoperative wound infections for use in clinical trials of antibiotic prophylaxis. Lancet.

[18] Abu-Sheasha GA, Bedwani RN, Anwar MM, Yassine OG (2020). Cost-effectiveness analysis of three methods of surgical-site infection surveillance: less is more. Am. J. Infect. Control.

[19] Aiken AM (2013). Evaluation of surveillance for surgical site infections in Thika Hospital, Kenya. J. Hospital Infect..

[20] Bluebelle Study Group. Validation of the Bluebelle Wound Healing Questionnaire for assessment of surgical-site infection in closed primary wounds after hospital discharge. *Br. J. Surg.* **106**, 226–235 (2019). 10.1002/bjs.11008 PMC6457211 30556594

[21] Costa ML (2020). Effect of incisional negative pressure wound therapy vs standard wound dressing on deep surgical site infection after surgery for lower limb fractures associated with major trauma: the WHIST randomized clinical trial. Jama.

[22] Cherian T (2020). Diagnosing post-cesarean surgical site infections in rural Rwanda: development, validation, and field testing of a screening algorithm for use by community health workers. Surg. Infect..

[23] Gunter RL (2018). Feasibility of an image-based mobile health protocol for postoperative wound monitoring. J. Am. Coll. Surg..

[24] Hedrick TL (2015). Defining surgical site infection in colorectal surgery: an objective analysis using serial photographic documentation. Dis. Colon Rectum.

[25] McLean KA (2021). Remote diagnosis of surgical-site infection using a mobile digital intervention: a randomised controlled trial in emergency surgery patients. npj Digital Med..

[26] Mitchell DH, Swift G, Gilbert GL (1999). Surgical wound infection surveillance: the importance of infections that develop after hospital discharge. Aust. N. Z. J. Surg..

[27] Nguhuni B (2017). Reliability and validity of using telephone calls for post-discharge surveillance of surgical site infection following caesarean section at a tertiary hospital in Tanzania. Antimicrob. Resist Infect. Control.

[28] Pathak A, Sharma S, Sharma M, Mahadik VK, Lundborg CS (2015). Feasibility of a mobile phone-based surveillance for surgical site infections in rural India. Telemed. J. E Health.

[29] Pham JC, Ashton MJ, Kimata C, Lin DM, Nakamoto BK (2016). Surgical site infection: comparing surgeon versus patient self-report. J. Surg. Res.

[30] Reilly J (2005). A study of telephone screening and direct observation of surgical wound infections after discharge from hospital. J. Bone Jt. Surg. Br. Vol..

[31] Richter V, Cohen MJ, Benenson S, Almogy G, Brezis M (2017). Patient self-assessment of surgical site infection is inaccurate. World J. Surg..

[32] Sands K, Vineyard G, Platt R (1996). Surgical site infections occurring after hospital discharge. J. Infect. Dis..

[33] Taylor EW (2003). Telephone call contact for post-discharge surveillance of surgical site infections. A pilot, methodological study. J. Hospital Infect..

[34] Halwani MA, Turnbull AE, Harris M, Witter F, Perl TM (2016). Postdischarge surveillance for infection following cesarean section: a prospective cohort study comparing methodologies. Am. J. Infect. Control.

[35] Mousa AY (2019). Results of telehealth electronic monitoring for post discharge complications and surgical site infections following arterial revascularization with groin incision. Ann. Vasc. Surg..

[36] Zhang J (2021). Wound image quality from a mobile health tool for home-based chronic wound management with real-time quality feedback: randomized feasibility atudy. JMIR Mhealth Uhealth.

[37] Wilson J (2017). Preventing surgical site infection: the challenge of ‘getting it right first time’. J. Infect. Prev..

[38] Singh S, Davies J, Sabou S, Shrivastava R, Reddy S (2015). Challenges in reporting surgical site infections to the national surgical site infection surveillance and suggestions for improvement. Ann. R. Coll. Surg. Engl..

[39] Tennison I (2021). Health care’s response to climate change: a carbon footprint assessment of the NHS in England. Lancet Planet Health.

[40] Bloomfield PS, Clutton-Brock P, Pencheon E, Magnusson J, Karpathakis K (2021). Artificial intelligence in the NHS: climate and emissions. J. Clim. Change Health.

[41] Savji N (2013). Association between advanced age and vascular disease in different arterial territories: a population database of over 3.6 million subjects. J. Am. Coll. Cardiol..

[42] Lam K, Lu AD, Shi Y, Covinsky KE (2020). Assessing telemedicine unreadiness among older adults in the United States during the COVID-19 pandemic. JAMA Intern. Med..

[43] Elbaz, S. et al. A systematic review of telemedicine for older adults with dementia during COVID-19: an alternative to in-person health services? *Front. Neurol.* **12**, 10.3389/fneur.2021.761965 (2021). 10.3389/fneur.2021.761965 PMC8712684 34970210

[44] Wilson AP (2004). Surgical wound infection as a performance indicator: agreement of common definitions of wound infection in 4773 patients. BMJ.

[45] Campwala I, Unsell K, Gupta S (2019). A comparative analysis of surgical wound infection methods: predictive values of the CDC, ASEPSIS, and Southampton scoring systems in evaluating breast reconstruction surgical site infections. Plast. Surg. (Oakv.).

[46] Moher D (2015). Preferred reporting items for systematic review and meta-analysis protocols (PRISMA-P) 2015 statement. Syst. Rev..

[47] Deeks J. B. P., Leeflang M., Takwoingi Y., Flemyng E., Mellor L. Cochrane Handbook for Systematic Reviews of Diagnostic Test Accuracy, https://training.cochrane.org/handbook-diagnostic-test-accuracy/PDF/v2 (2021). 10.1002/14651858.ED000163 PMC10408284 37470764

[48] McInnes MDF (2018). Preferred reporting items for a systematic review and meta-analysis of diagnostic test accuracy studies: the PRISMA-DTA atatement. JAMA.

[49] Lathan R. et al. Telemedicine for surgical site infection: a systematic review. https://www.crd.york.ac.uk/prospero/display_record.php?ID=CRD42021290610 (2021).

[50] Horan TC, Gaynes RP, Martone WJ, Jarvis WR, Emori TG (1992). CDC definitions of nosocomial surgical site infections, 1992: a modification of CDC definitions of surgical wound infections. Infect. Control Hosp. Epidemiol..

[51] Ouzzani M, Hammady H, Fedorowicz Z, Elmagarmid A (2016). Rayyan—a web and mobile app for systematic reviews. Syst. Rev..

[52] Whiting PF (2011). QUADAS-2: a revised tool for the quality assessment of diagnostic accuracy studies. Ann. Intern Med.

[53] Freeman SC (2019). Development of an interactive web-based tool to conduct and interrogate meta-analysis of diagnostic test accuracy studies: MetaDTA. BMC Med. Res. Methodol..

[54] Review Manager (RevMan). Version 5.4 (The Cochrane Collaboration, 2020).

